# Pervasive Transcription of the Human Genome Produces Thousands of Previously Unidentified Long Intergenic Noncoding RNAs

**DOI:** 10.1371/journal.pgen.1003569

**Published:** 2013-06-20

**Authors:** Matthew J. Hangauer, Ian W. Vaughn, Michael T. McManus

**Affiliations:** Diabetes Center, Department of Microbiology and Immunology, University of California, San Francisco, California, United States of America; Broad Institute of MIT and Harvard, United States of America

## Abstract

Known protein coding gene exons compose less than 3% of the human genome. The remaining 97% is largely uncharted territory, with only a small fraction characterized. The recent observation of transcription in this intergenic territory has stimulated debate about the extent of intergenic transcription and whether these intergenic RNAs are functional. Here we directly observed with a large set of RNA-seq data covering a wide array of human tissue types that the majority of the genome is indeed transcribed, corroborating recent observations by the ENCODE project. Furthermore, using *de novo* transcriptome assembly of this RNA-seq data, we found that intergenic regions encode far more long intergenic noncoding RNAs (lincRNAs) than previously described, helping to resolve the discrepancy between the vast amount of observed intergenic transcription and the limited number of previously known lincRNAs. In total, we identified tens of thousands of putative lincRNAs expressed at a minimum of one copy per cell, significantly expanding upon prior lincRNA annotation sets. These lincRNAs are specifically regulated and conserved rather than being the product of transcriptional noise. In addition, lincRNAs are strongly enriched for trait-associated SNPs suggesting a new mechanism by which intergenic trait-associated regions may function. These findings will enable the discovery and interrogation of novel intergenic functional elements.

## Author Summary

Much of the human genome is composed of intergenic sequence, the regions between genes. Intergenic sequence was once thought to be transcriptionally silent “junk DNA,” but it has recently become apparent that intergenic regions can be transcribed. However, the scope, nature, and identity of this intergenic transcription remain unknown. Here, by analyzing a large set of RNA-seq data, we found that >85% of the genome is transcribed, allowing us to generate a comprehensive catalog of an important class of intergenic transcripts: long intergenic noncoding RNAs (lincRNAs). We found that the genome encodes far more lincRNAs than previously known. A key question in the field is whether these intergenic transcripts are functional or transcriptional noise. We found that the lincRNAs we identified have many characteristics that are inconsistent with noise, including specific regulation of their expression, the presence of conserved sequence and evidence for regulated processing. Furthermore, these lincRNAs are strongly enriched with intergenic sequences that were previously known to be functional in human traits and diseases. This study provides an essential framework from which the functional elements in intergenic regions can be identified and characterized, facilitating future efforts toward understanding the roles of intergenic transcription in human health and disease.

## Introduction

A large fraction of the human genome consists of intergenic sequence. Once referred to as “junk DNA”, it is now clear that functional elements exist in intergenic regions. In fact, genome wide association studies have revealed that approximately half of all disease and trait-associated genomic regions are intergenic [1]. While some of these regions may function solely as DNA elements, it is now known that intergenic regions can be transcribed [2]–[7], and a growing list of functional noncoding RNA genes within intergenic regions has emerged [8].

Despite this progress, a complete understanding of the extent of intergenic transcription and the identity of these transcripts has remained elusive. The first attempts to analyze the extent and nature of intergenic transcription utilized tiling array technology [2]–[5]. These studies suggested that intergenic transcription is pervasive, but concerns about cross-hybridization have fueled a debate about the data [9]–[12]. Furthermore, in order to avoid technical difficulties associated with analyzing repeat sequence using tiling arrays, the studies were restricted to evaluating less than half of the genome. More recently, a few studies have focused on evaluating the extent of intergenic transcription using sequencing-based approaches, but with the exception of the recently published ENCODE project results [13], [14], these studies have thus far been limited to very narrow preselected regions of the genome and a small number of tissues [6], [7]. Overcoming these prior shortcomings, the ENCODE project used RNA-seq analysis in combination with other technologies to profile 15 human cell lines, providing evidence for transcription across 83.7% of the human genome and firmly establishing the reality of pervasive transcription [14].

Long intergenic noncoding RNAs (lincRNAs) are defined as intergenic (relative to current gene annotations) transcripts longer than 200 nucleotides in length that lack protein coding capacity. LincRNAs are known to perform myriad functions through diverse mechanisms ranging from the regulation of epigenetic modifications and gene expression to acting as scaffolds for protein signaling complexes [8], [15]. The first attempts to generate lincRNA annotation sets either profiled lincRNAs specific to a small number of tissues or required that transcripts harbor specific structural features such as splicing and polyadenylation [16]–[18]. The GENCODE consortium (GENCODE v7) has manually curated approximately five thousand lincRNAs that are not restricted to particular tissues or structural features, however this annotation set contains only a small fraction of all lincRNAs because it does not take advantage of RNA-seq data to identify novel transcripts [19], [20]. The limited scale of current lincRNA annotations, including GENCODE, is clearly incompatible with the massive amount of intergenic transcription observed by the ENCODE project. It should therefore be expected that the genome encodes far more lincRNAs than are currently known.

In order to bridge the gap between the observation of pervasive intergenic transcription by the ENCODE project and the currently limited set of annotated lincRNAs, we performed an analysis of a unique set of RNA-seq data derived from both novel and published datasets that complements and significantly expands prior efforts [14], [16], [19]. This analysis resulted in a clear corroboration of the observations of pervasive transcription across the human genome by the ENCODE project [14]. Furthermore, analysis of previously annotated putative lincRNAs, including those of the ENCODE project [19], in addition to *de novo* discovery of novel lincRNAs from RNA-seq data has resulted in the compilation of the most comprehensive catalog of human lincRNAs. Owing to the extended breadth of tissues sampled and relaxed constraints on transcript structure, we find significantly more lincRNAs than all previous lincRNA annotation sets combined. Our analyses revealed that these lincRNAs display many features consistent with functionality, contrasting prior claims that intergenic transcription is primarily the product of transcriptional noise [12]. In sum, our findings corroborate recent reports of pervasive transcription across the human genome and demonstrate that intergenic transcription results in the production of a large number of previously unknown lincRNAs. We provide this vastly expanded lincRNA annotation set as an important resource for the study of intergenic functional elements in human health and disease.

## Results

### Quantitation of the Extent of Transcription of the Human Genome

**Figure 1:**
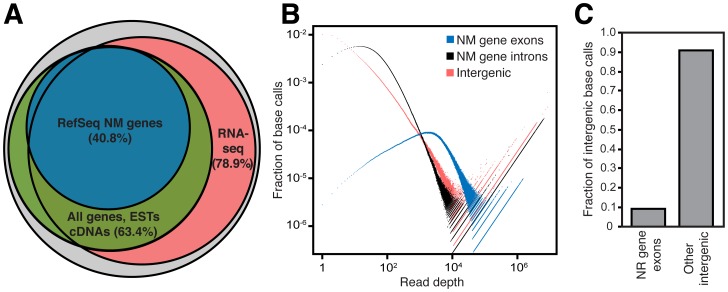
The human intergenic transcriptome. (A) 85.2% of the genome has evidence of transcription, with RNA-seq reads mapping directly to 78.9% of genomic sequence. The remaining genomic coverage is comprised of known genes, spliced ESTs and spliced cDNAs. The grey circle represents the portion of the genome (83.4%) that is uniquely mappable with RNA-seq reads. (B) Protein coding (NM gene) exon, intron and intergenic region expression level distribution. Regions that have high expression have a larger fraction of base calls appearing at higher read depths. Protein coding gene exons have the highest proportion of bases with high read depth, while introns and intergenic regions have relatively more bases of low read depth though each contain many highly expressed regions. Base calls = (# of genomic positions at a specific read depth)(read depth). (C) Most intergenic transcription is outside of annotated noncoding RNA genes. The fraction of intergenic base calls within RefSeq noncoding RNA genes (NR genes) compared to other intergenic regions are compared. In (A–C), only uniquely mappable portions of the genome are considered (see Methods).

We have analyzed six novel RNA-seq datasets generated as part of the Human Epigenome Atlas (http://www.genboree.org/epigenomeatlas/index.rhtml) and 121 previously published RNA-seq datasets representing 23 human tissues under multiple conditions and consisting of over 4.5 billion uniquely mapped reads (Table S1). This set of RNA-seq data allowed for detection of both rare and tissue-specific transcription events that would otherwise be undetectable. In contrast to the limited reach of prior tiling array studies [2]–[5], we analyzed the much larger portion (83.4%) of the genome to which RNA-seq reads can be uniquely mapped thus providing a broader view of the transcriptome. At a threshold of one RNA-seq read, we observed reads mapping to 78.9% of the genome and, if additional evidence of transcription is taken into account including the full structures of known genes, spliced ESTs and cDNAs, we found evidence that 85.2% of the genome is transcribed (Figure 1A). This result closely agrees with the recently published findings from the ENCODE project in which evidence for transcription of 83.7% of the genome was uncovered [14]. Interestingly, even with 4.5 billion mapped reads, we observe an increase in genomic coverage at each lower read threshold implying that even more read depth may reveal yet higher genomic coverage. (Figure S1).

As expected, protein coding gene exons contain the largest fraction of highly expressed bases (Figure 1B) as well as a disproportionately large fraction of total reads relative to their small (<3%) amount of genomic sequence (Figure S2). However, many regions of high expression do exist within intergenic regions, far more than are accounted for by current noncoding RNA gene annotations (Figure 1C). We reasoned that this unaccounted for intergenic transcription must derive from novel intergenic transcripts, and we next directed our efforts toward identification and analysis of these transcripts.

### Discovery of a Large Number of Novel LincRNAs

We hypothesized that much of the intergenic transcription not accounted for by previously annotated transcripts is derived from novel lincRNAs. We reasoned that because lincRNA expression is known to be highly tissue-specific [16], the breadth of tissues and conditions sampled in the RNA-seq datasets analyzed here would aid lincRNA discovery.

**Figure 2:**
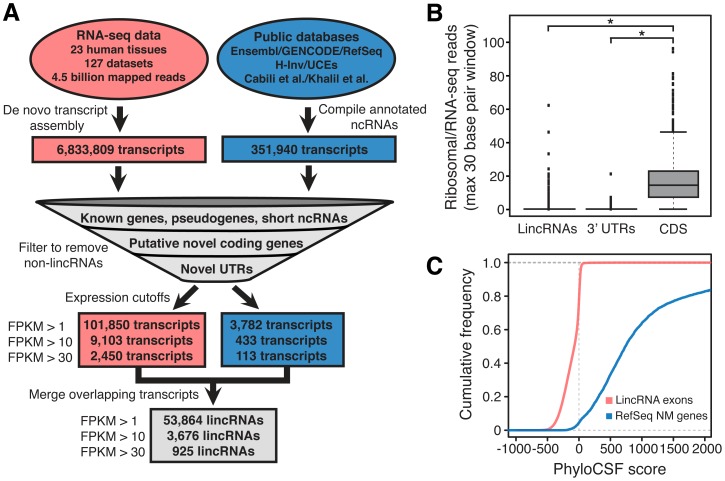
Discovery of lincRNAs. (A) Discovery of lincRNAs consisted of *de novo* assembly of transcripts from RNA-seq data and compilation of annotated and putative noncoding RNAs (see Methods), followed by a series of filters designed to remove all known and novel protein coding transcripts and non-lincRNA noncoding RNAs. Only intergenic noncoding transcripts at least 200 nucleotides in length and expressed at least at one copy per cell were ultimately annotated as lincRNAs. (B) Analysis of ribosomal profiling data reveals that the lincRNA catalog is composed of noncoding transcripts. The maximum 30 bp window ratio of HeLa ribosomal/RNA-seq reads [22] is plotted for exons of lincRNAs, 3′ UTRs and coding sequences (CDS). *P<2.2E-16; whiskers extend +/−1.5 times interquartile range and dots represent outliers. (C) Computational analysis of protein coding capacity of the lincRNAs reveals a lack of protein coding capacity. The cumulative distribution of PhyloCSF [40] scores for lincRNAs and RefSeq NM genes are plotted. Higher scores correspond to higher predicted coding capacity.

We used this large set of RNA-seq data in combination with previous noncoding RNA annotation sets to generate the most comprehensive catalog of lincRNAs (Figure 2A). In order to generate this lincRNA catalog, we first compiled known and putative annotated lincRNAs. We collected noncoding RNAs present in public databases, including GENCODE v6, and from literature sources [16], [18] resulting in a set of 351,940 transcripts. In addition, we performed *de novo* transcriptome assembly on each of the RNA-seq datasets (Table S2) to generate 6,833,809 *de novo* assembled transcripts. Both previously annotated and *de novo* assembled transcripts were filtered to remove transcripts overlapping protein coding genes, known non-lincRNA noncoding RNA genes, and pseudogenes. Transcripts longer than 200 nucleotides were further filtered to remove any transcripts containing (or overlapping any other transcript containing) an open reading frame (ORF) longer than 100 amino acids. Out of concern that some *de novo* assembled transcripts may be unannotated extensions of neighboring protein coding genes, as was recently observed for a fraction of GENCODE long noncoding RNAs [19], we created an additional filter to remove transcripts linked to neighboring genes by RNA-seq reads. To do this, we extended protein coding gene reference annotations using *de novo* transcriptome assembly and removed transcripts overlapping these extended gene structures (see Methods, Dataset S1).

In a final step, we removed transcripts expressed at fragments per kilobase of transcript per million mapped reads (FPKM)<1, a threshold approximately equivalent to one copy per cell [21] (Table S1). To decrease redundancy, and with the goal of identifying lincRNA “genes” rather than potentially redundant overlapping “transcripts”, the remaining transcripts were merged if they shared at least one exon (see Methods) resulting in 53,864 distinct putative lincRNAs at FPKM>1, 3,676 lincRNAs at FPKM>10, and 925 lincRNAs at FPKM>30 (Dataset S2 and Figure S3). Surprisingly, greater than 94% of the final set of merged lincRNAs at each expression level consists exclusively of novel *de novo* assembled transcripts discovered from the RNA-seq data in this study (Table S3 and Dataset S2). Rather than being clustered near currently annotated genes, these lincRNAs are spread throughout intergenic sequence. 58.1% of FPKM>1 lincRNAs, 61.9% of FPKM>10 lincRNAs, and 67.7% of FPKM>30 lincRNAs are greater than 30 kilobases from the nearest protein coding gene on either strand. We annotated the lincRNAs as belonging to the same “group” (see Methods) if they are within 1 kilobase of each other to account for the possibility that some proximal lincRNA annotations may be partial structures of larger transcripts (see Discussion). This grouping resulted in 35,585 distinct lincRNA groups at FPKM>1, 2,970 at FPKM>10, and 764 at FPKM>30, and the lincRNAs in the catalog are named according to these groups (Dataset S2). These annotations are likely to be incomplete due to limitations in transcript assembly from RNA-seq data; indeed, some annotations may be fragments of larger overlapping lincRNA transcripts. Therefore, the actual number of independent lincRNAs may differ from the above numbers, and future work is needed to more fully define complete, independent lincRNA transcript annotations (see Discussion).

### Evaluation of LincRNA Filtering Approach

We evaluated the stringency with which our filtering process removed protein coding transcripts by analyzing ribosomal profiling data from HeLa cells (Figure 2B) [22]. As expected, lincRNAs resemble the 3′ untranslated region exons of protein coding genes, with very few transcripts showing significant engagement with the ribosome. This finding is in agreement with the recent observation that GENCODE long noncoding RNAs (a subset of our catalog) generally lack mass spectrometry based evidence for translation [23]. In contrast, a recent study found that many previously annotated mouse lincRNAs bind the ribosome [24]. While the biological significance of this discrepancy is unknown, it may be the result of differences in the stringency of the filtering approach employed in the generation of the lincRNA annotations under consideration. Further confirming the stringency of our filters, a computational analysis of protein coding potential using the program PhyloCSF revealed that our set of filtered lincRNAs lack predicted protein coding capacity (Figure 2C). From these analyses we conclude that our filtering approach effectively removed protein coding transcripts from the catalog.

### Additional LincRNA Catalogs and Resources

While the remainder of this study focuses on this catalog of putative lincRNAs (Dataset S2), we have provided multiple alternative lincRNA catalogs. These include a combined catalog of the lincRNAs identified in this study merged (see Methods) with a set of additional lincRNAs identified in Cabili, *et al.* [16] which passed all of our filters except were not expressed at FPKM>1 in any of the RNA-seq datasets analyzed here. The added lincRNAs are expressed at FPKM>1 in one or more of the RNA-seq datasets analyzed in Cabili *et al.* [16], which are entirely distinct from the datasets analyzed here, and are therefore likely to be genuine lincRNAs by our criteria. This catalog (Dataset S3) includes 54,784 lincRNAs at FPKM>1 (920 additional lincRNAs compared to Dataset S2), 3,764 lincRNAs at FPKM>10 (88 additional lincRNAs), and 942 lincRNAs at FPKM>30 (17 additional lincRNAs). In addition, we have included a catalog of spliced lincRNAs that are expressed at FPKM>1 in at least one dataset (4,576 lincRNAs, Dataset S4), of which 61% are exclusively composed of *de novo* assembled transcripts discovered in this study. We have also compiled a catalog of lincRNAs expressed at FPKM>1 in at least two datasets (26,455 lincRNAs, Dataset S5), of which 97% are exclusively *de novo* assembled transcripts discovered here. Additionally, an alternative lincRNA catalog containing only those lincRNAs expressed significantly higher than randomly sampled intergenic regions (see Methods) were included (5,267 lincRNAs, Datasets S6, S7). Furthermore, as an additional resource we provide the expression level (FPKM and raw RNA-seq read counts) of all lincRNAs (in Dataset S2) and RefSeq protein coding genes across all 127 RNA-seq datasets (Dataset S8).

### LincRNAs Are Specifically Regulated

**Figure 3:**
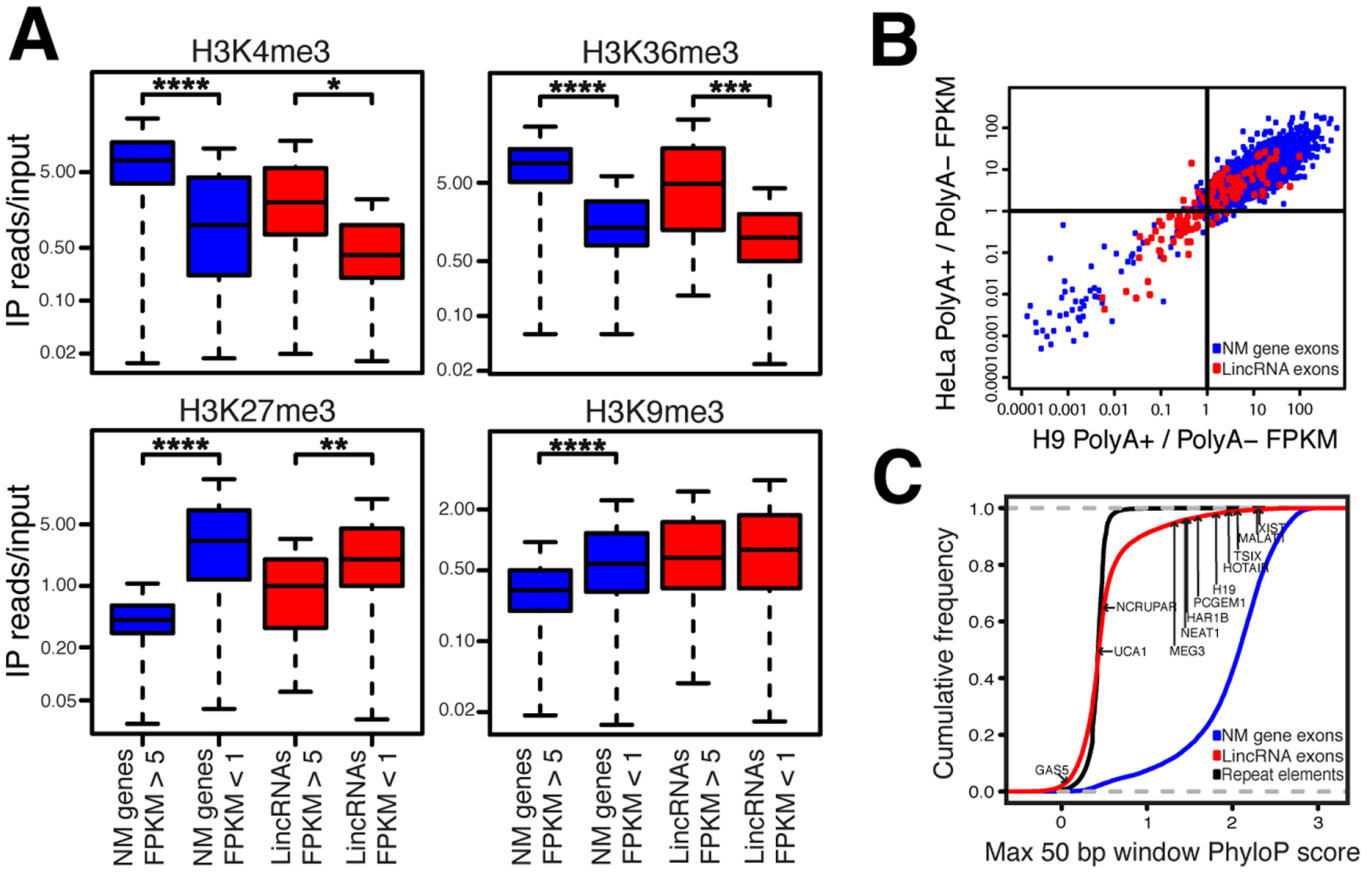
LincRNAs possess features inconsistent with transcriptional noise. (A) ChIP-seq and RNA-seq data from IMR90 cells [28], [29] were analyzed for lincRNAs and RefSeq NM genes. *P = 4.01E-7, ** *P* = 4.52E-9, *** *P* = 2.43E-14, **** *P*<2.2E-16; *P* = 0.137 for lincRNAs H3K9me3; whiskers extend to +/−1.5 times interquartile range or most extreme data point. (B) LincRNA FPKM values in polyA+ specific and polyA− specific RNA-seq libraries in H9 ESCs and HeLa cells [46] were compared. Transcripts with RNA-seq reads in all four datasets and with FPKM>1 in at least one of the two fractions for each cell type were analyzed (16,819 NM genes and 127 lincRNAs). Individual lincRNA and NM gene ratios of FPKMs in polyA+/polyA− fractions are plotted. Pearson correlation value for lincRNAs = 0.622 (*P* = 5.551E-15) and for NM genes = 0.702 (*P*<2.2E-16). (C) The maximally conserved 50 bp windows in each NM gene, lincRNA, and repetitive element (nonconserved control sequences) were determined. The maximally conserved 50 bp windows of 12 functional human lincRNAs are indicated for comparison.

The degree to which intergenic transcription is functional remains uncertain and controversial [9]–[12], [25]. In order to evaluate whether the lincRNAs identified in the present study are specifically regulated as opposed to transcriptional noise, we determined if the lincRNA genes harbor canonical epigenetic marks for activation and repression with the reasoning that noise transcripts should lack coherent epigenetic modification patterns. Consistent with observations based on earlier long noncoding RNA annotations [18], [19], [26], [27], analysis of ChIP-seq and RNA-seq data [28], [29] revealed that the catalog of lincRNAs shows patterns of epigenetic modification similar to protein coding genes (Figure 3A). Activating histone marks, H3K4me3 and H3K36me3, are both significantly enriched within highly expressed lincRNAs. Similarly, the repressive mark H3K27me3 is significantly enriched within lowly expressed lincRNAs. Thus, the expression of lincRNAs appears to be specifically regulated.

If lincRNAs are specifically regulated at the level of transcription, it is expected that their expression levels are specific to their tissue source. Indeed, prior studies of lincRNAs have shown that lincRNAs display very strong tissue-specific expression [16], [19]. To test whether this remains true with our expanded set of lincRNAs we performed unsupervised hierarchical clustering using lincRNA expression levels in replicate RNA-seq datasets from various tissues (Figure S4). Replicates of each tissue type strongly clustered together, indicating that lincRNA differential expression is indeed reproducibly tissue-specific, supporting specific regulation of lincRNA expression.

LincRNAs do not need to be polyadenylated to be functional [30]. Because of this, we included in our analysis many RNA-seq libraries that were not polyA+ selected. In fact, earlier tiling array studies revealed that intergenic transcripts tend to be bimorphic; that is, they appear in both polyA+ and polyA− fractions, as opposed to protein coding transcripts that are primarily polyA+ [3]. The recently published ENCODE results corroborate this finding [14], [19]. In agreement with these studies, we found that the polyadenylation status of lincRNAs in our catalog is reproducibly bimorphic across multiple cell types while protein coding transcripts are strongly enriched in the polyA+ sample. The reproducibility of this lincRNA bimorphic state suggests that lincRNA polyadenylation is regulated and that many lincRNAs exist at least partially as nonpolyadenylated transcripts (Figure 3B and Figure S5). This finding indicates that future studies of lincRNAs should not ignore the nonpolyadenylated RNA fraction.

We next evaluated whether lincRNAs are conserved. It has been observed that lincRNAs can contain conserved motifs tethered together by nonconserved sequence [25], [31], [32]. Therefore, we evaluated lincRNA conservation using a scanning 50 bp window (Figure 3C, Figure S6, and Table S4). Consistent with prior studies, lincRNAs display detectable but modest conservation [16], [19]. We applied this same method to known functional human lincRNAs and found that the majority of the lincRNAs identified in this study display a level of conservation consistent with known functional lincRNAs (Figure 3C).

### LincRNAs Are Enriched for Trait-Associated SNPs

**Figure 4:**
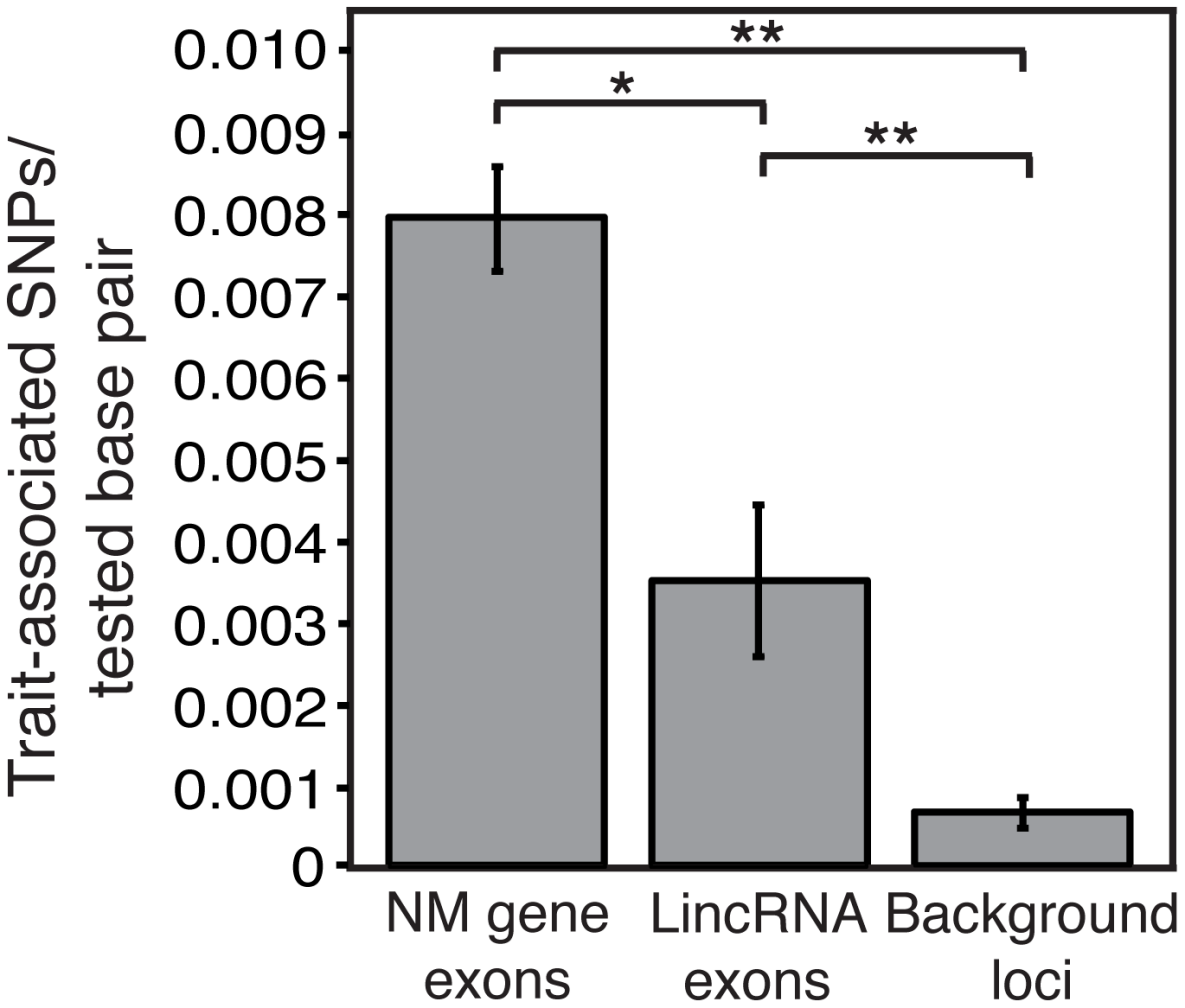
LincRNAs are enriched for trait-associated SNPs. The number of trait-associated SNPs within RefSeq NM gene exons, lincRNA exons, or background loci (nonexpressed intergenic sequence) per tested SNP in genome wide association studies is compared (see Methods). *P = 0.0173, **P<2.2E-16; error bars represent 95% binomial proportion confidence interval.

Almost half of all trait-associated SNPs (TASs) identified in genome-wide association studies are located in intergenic sequence while only a small portion are in protein coding gene exons [1]. This curious observation points to an abundance of functional elements in intergenic sequence. While some of these regions may function at the DNA level alone, it is possible that many function by encoding RNA. In fact, TASs have already been identified within or proximal to noncoding RNAs including some lincRNAs [16], [33]–[36]. We reasoned that if lincRNAs are functional, they should be enriched for TASs compared to nonexpressed intergenic regions. Indeed, we find that lincRNAs are more than 5-fold enriched for TASs compared to nonexpressed intergenic regions (Figure 4) despite an approximately equal distribution of SNPs between these regions (Figure S7). Therefore, many trait-associated intergenic regions may function by encoding lincRNAs.

## Discussion

There has been a recent debate about whether there is pervasive transcription of the human genome and what the number and abundance of intergenic transcripts is [9]–[12]. Until recently, a key missing component to this debate has been an analysis of ultra deep RNA-seq data sampling a wide array of tissue types. Without this, insufficient read depth can result in a failure to identify low abundance intergenic transcripts, and limited tissue sampling results in missed tissue specific expression. During the course of this study, the ENCODE project released a large scale analysis of RNA-seq data that provided clear evidence that the human genome is pervasively transcribed [14]. We analyzed a distinct, complementary set of RNA-seq data that also fulfills these requirements of read depth and tissue breadth, covering both polyadenylated and nonpolyadenylated RNA fractions. In strong agreement with the ENCODE results, we observed that approximately 85% of the genome is transcribed, supporting prior observations of pervasive transcription based on tiling arrays that have been recently questioned [2]–[5].

There is an apparent discrepancy between this observed pervasive transcription and the relative paucity of annotated lincRNAs, the most numerous intergenic RNAs. It should be expected that intergenic regions encode far more lincRNAs than are currently annotated. Indeed, here we found that there are many more lincRNAs than previously known, even after aggressive filtering that removed the vast majority of previously annotated long noncoding RNAs and newly discovered intergenic transcripts (Dataset S2). These observations clearly demonstrate that the human genome is pervasively transcribed, and that lincRNAs make up an extremely common class of intergenic transcripts.

In agreement with prior observations of smaller lincRNA annotation sets, our analyses of the expanded lincRNA catalog presented here revealed that most lincRNAs are expressed at lower levels than protein coding genes [16], [19]. Though most lincRNAs are expressed at only a few copies per cell, we found that many lincRNAs are highly expressed with nearly 4,000 expressed at >FPKM 10 and nearly 1,000 expressed at >FPKM 30, rivaling the expression of many messenger RNAs. We chose to apply an expression cutoff to remove very lowly expressed transcripts from the catalog of lincRNAs. However, it may be the case that there exist many functional lincRNAs with very low expression levels, below our expression filter cutoff. For example, the functional human lincRNA HOTTIP is expressed in approximately one out of three cells [37]. Furthermore, recent findings have shown that the intergenic transcriptome may be vastly more complex than currently appreciated when very lowly expressed transcripts are considered [7]. It is possible that some of these are functional transcripts despite their apparent low expression, perhaps having brief bursts of expression during stages of the cell cycle or functioning in single cells in a heterogeneous population as has been previously observed [14]. Therefore, while we have provided the most complete lincRNA catalog to date, there may be additional lowly expressed, yet potentially functional lincRNAs that were excluded here.

In order to minimize any potential contamination of the lincRNA catalog with protein coding transcripts, the filtering approach used was very aggressive. In fact, most previously annotated noncoding RNAs failed to pass our filters and were therefore excluded from the lincRNA catalog (Table S3 and Dataset S9). The vast majority of these transcripts (including most GENCODEv6 “lincRNAs” and “processed transcripts”) overlap known or predicted protein coding genes, pseudogenes, or non-lincRNA noncoding RNAs (e.g. microRNAs)(Table S3). Some of these removed transcripts may be functional long noncoding RNAs, such as GAS5 (removed because it contains 10 snoRNA genes within its introns). However, in order to most confidently identify only lincRNAs, rather than potential unannotated extensions of known genes, these were removed.

Of those previously annotated noncoding RNAs that are intergenic, more than half contain predicted ORFs longer than 100 amino acids. For example, two previously characterized functional human lincRNAs were found to contain ORFs longer than 100 amino acids, Xist and HOTAIR. These results demonstrate that our filtering approach, which eliminates all transcripts with ORFs larger than 100 amino acids, may have removed some lincRNAs with large, nonfunctional ORFs. However, the use of a 100 amino acid ORF cutoff, a commonly used threshold to define potential protein coding genes, is justifiable because ORFs of this size infrequently occur by chance and instead indicate potential for protein coding capacity [38], [39].

Rather than discard all transcripts with large ORFs, as we did here, one option to discriminate between transcripts that are coding versus noncoding is to analyze the frequency of synonymous codon substitutions (PhyloCSF) [40]. However, this approach is limited to ORFs that can be aligned across species, potentially missing recently evolved or otherwise nonconserved novel protein coding genes. Importantly, our approach of removing all transcripts with large open reading frames effectively removed transcripts with significant predicted coding potential (Figure 2C), indicating that using an ORF size cutoff is at least as conservative as filtering based on PhyloCSF analysis. The lack of engagement of the ribosome, observed with ribosomal profiling data, confirms the stringency of the ORF cutoff filter (Figure 2B). Further analysis of these removed large ORF-containing intergenic transcripts is outside the scope of this study, but we have included these annotations for investigators interested in further analyzing their coding potential in search of novel protein coding genes (Dataset S10).

Despite the fact that most previously annotated noncoding RNAs failed to pass our filters, our lincRNA catalog contains significantly more lincRNAs than previously known (>94% of lincRNAs are entirely novel at each expression level). This is the result of two unique features of our study. First, the RNA-seq read depth and diversity of tissues surveyed allowed for the detection of rare and tissue specific transcripts that were previously unknown. Many of these novel transcripts passed all filters and are annotated as novel lincRNAs in our catalog. Second, in contrast to prior lincRNA annotation efforts that were restricted to identification of only spliced or polyadenylated lincRNAs [16], [19], [41], we sought to generate annotations of a more complete set of human lincRNAs regardless of splicing or polyadenylation status. The reasons for taking this approach are manifold. Two of the most well known and abundant functional human lincRNAs, NEAT1 and MALAT1, are single exon genes (as are approximately 5% of protein coding genes) [42], suggesting that non-spliced transcripts may make up an important class of lincRNA. Additionally, numerous functional nonpolyadenylated noncoding RNAs have been described [30], [43]. Even long noncoding RNAs which can be spliced are often found in their unprocessed forms [44], a distinct property of long noncoding RNAs that would result in missed lincRNAs if splicing were a required attribute. Therefore, we chose not to exclude any lincRNAs from this catalog due to lack of splicing or polyadenylation. Importantly, because nonspliced, nonpolyadenylated transcripts could theoretically be erroneously *de novo* assembled from reads derived from contaminating genomic DNA in RNA-seq data, we took multiple measures to mitigate any contributions of genomic DNA contaminant reads (see Methods).

Due to inherent limitations of *de novo* transcriptome assembly using short reads of finite depth, it is not always possible to unequivocally determine the complete structure of a transcript. This is particularly true for lowly expressed transcripts where the number of reads available is limited, and for genomic regions to which reads cannot be uniquely mapped. In the case of shallow read depth, exons of multi-exonic transcripts may lack reads connecting the exons, and *de novo* assembly could result in separate annotation of each exon as a distinct transcript. In support of this, we found that lower expressed lincRNAs discovered from *de novo* transcript assembly were less likely to have multi-exonic structures (Table S5). Additionally, the annotated 5′ and 3′ ends of the lincRNAs may represent truncations of the full length transcripts. Indeed, our analysis of PET tag data revealed that while the majority of our lincRNA catalog is overlapped by at least one PET tag, in most cases there is minimal PET tag support for the annotated 5′ and 3′ ends of the lincRNAs (Table S6). It is therefore the case that some lincRNA annotations in the catalog we provide (Dataset S2), particularly single exon lincRNA annotations, may represent fragments of larger transcripts.

Furthermore, considering the reported prevalence of low level overlapping transcripts throughout intergenic sequence [7], it is not clear that full lincRNA structures can be unequivocally deconvoluted using short read RNA-seq technology. The determination of full lincRNA structures will be an important future effort in the field and may rely upon new datasets of longer read length and greater read depth, use of multiple orthogonal data types in the same tissue, new technologies such as ultra long read next generation sequencing, and further improvements in software for *de novo* transcript assembly.

In addition, the majority of RNA-seq data we analyzed lacks strand information and as a result most of the lincRNAs in our catalog are of ambiguous strandedness. Prior annotations have relied upon splice site orientation to infer the strandedness of the transcript [16]. While this is a reasonable approach that we too have adopted when applicable in the present lincRNA catalog, stranded RNA-seq data is needed to most confidently assign strandedness to *de novo* assembled transcripts.

While determining the isoforms and full structures of all lincRNAs is clearly desirable, these incomplete lincRNA structure annotations are nonetheless of tremendous practical value. Knowledge of the structure of a portion of a transcript is often sufficient to test for differential expression or perform RNAi knockdown experiments, and facilitates the cloning and sequencing of the full length transcript. Because of this, instead of placing additional restrictions upon lincRNA annotations, our filtering strategy was aimed toward identification of as many transcripts as possible that fit within the definition of a lincRNA. However, for investigators interested in more refined lincRNA annotations, we have provided multiple more restrictive lincRNA catalogs (Datasets S4, S5, S6).

A key question in the field is whether the transcripts resulting from pervasive transcription of intergenic regions are functional or the result of noisy transcription. The lincRNAs we describe are specifically regulated and contain conserved sequence, attributes inconsistent with transcriptional noise (Figure 3). Furthermore, lincRNAs were found to be strongly enriched for intergenic TASs compared to nonexpressed intergenic regions (Figure 4). This striking finding supports the possibility that many intergenic SNPs mark regions that function as lincRNAs rather than DNA elements. Because nearly half of all TASs are intergenic, it is possible that lincRNAs play a significant role in the majority of human traits and diseases thus far analyzed in GWASs. One functional lincRNA (MIAT) was first identified during the experimental interrogation of an intergenic TAS [35], and another lincRNA PTCSC3, was identified nearby a TAS found from a papillary thyroid carcinoma GWAS, perhaps representing the first of many such discoveries to come from intergenic TASs. The finding that lincRNAs are strongly enriched for TASs provides a new opportunity to revisit intergenic trait-associated regions with unknown functional mechanisms by testing whether the overlapping lincRNA is involved in the observed phenotype.

This noncoding RNA catalog represents a major step toward achieving a more complete understanding of this exciting frontier. We have identified a large number of putative lincRNAs with characteristics suggesting functionality. However, many of these lincRNAs are low expressed and definitive proof of functionality for a lincRNA requires functional experiments. High throughput functional genomic approaches, such as RNAi and cDNA overexpression screens, will serve as crucial tools for future efforts to uncover the roles of lincRNAs in diverse biological systems. With the requisite technology now available for these next generation experimental approaches, the time is ripe for this dark matter of the human genome to step further into the spotlight.

## Materials and Methods

### RNA-seq and Ribosomal Profiling Read Alignment and Processing

127 RNA-seq sequence files (5 novel and 122 publicly available datasets, Table S1) were aligned to hg18 with TopHat v1.1.4 allowing only uniquely mapped reads using the option -g 1 (all other parameters were default, see the TopHat manual http://tophat.cbcb.umd.edu/manual.html). Detailed information pertaining to each dataset, including novel datasets, is available in the sources provided in Table S1. These RNA-seq datasets were chosen because they sampled a wide breadth of human tissues and cell types, have well documented experimental methods used for their generation, and were publicly available. While datasets with longer reads and deeper read depth were preferred because they allow for more complete *de novo* transcript assembly, some datasets with short reads and shallow read depths were included in order to sample as many tissue types as possible. Datasets derived from tissues with mutated genomes, such as cancers, were included to capture tissue specific expression even though some reads from mutated genomic positions would fail to map to the reference hg18 genome. SAMtools v0.1.7 and BEDTools v2.12.0 were used to process aligned read files.

### Quantitation of the Transcribed Fraction of the Genome

The uniquely mappable human genome, defined here as the portions of the genome to which RNA-seq reads can be uniquely mapped, was derived for hg18 from http://www.imagenix.com/uniqueome/downloads/hg18_uniqueome.unique_starts.base-space.50.2.positive.BED.gz [45]. It contains 2,570,174,327 bp or 83.4% of the total human genomic sequence. To determine the genomic coverage of RNA-seq data, all aligned RNA-seq reads were combined and read coverage at each genomic base position was determined with the BEDTools function genomeCoverageBed. Split reads (i.e. exon-exon junction spanning reads) were counted such that intronic sequence was included as part of the reads. In Figure 1A, “All genes, ESTs, cDNAs” includes GENCODE v10 genes (excluding pseudogenes), RefSeq NM and NR genes, UCSC Known Genes, spliced H-Invitational cDNAs, spliced ESTs (UCSC Genome Browser “Spliced EST” track), and previously annotated spliced lincRNAs [16]. In all cases, intronic sequences of genes, cDNAs and ESTs were included.

### LincRNA Discovery

#### Transcripts annotated in public databases and literature sources that could be lincRNAs were compiled

Ensembl v61 “processed_transcript” and “lincRNA” categories, GENCODE v6 “processed_transcript” and “lincRNA” categories, RefSeq NR and XR genes, H-Invitational “noncoding” transcripts, ultra conserved elements (UCEs), and published lincRNAs from Khalil *et al.* [18] and Cabili *et al.* [16]. LiftOver was used to map hg19 coordinates to hg18 for Ensembl, GENCODE, H-Invitational and Cabili *et al.* [16] transcripts. RefSeq XR sequences in hg19 were aligned to hg18 with BLAT v34 and the top scoring alignment was used. Ultra conserved elements sequences were retrieved from http://biodev.cbm.fvg.it, aligned to hg18 with BLAT v34 and the top scoring alignment was used. Khalil *et al.* [18] exons were grouped by their overlapping defined transcribed regions to build transcript structures.

#### Novel transcripts from *de novo* transcriptome assembly of RNA-seq data were compiled

*De novo* transcriptome assembly was performed on RNA-seq data with Cufflinks v1.0.1 using the upper quartile normalization (-N) and fragment bias correction (-b) options. This transcript assembly was performed using reads that were prealigned to hg18 using TopHat as described above. In cases where multiple datasets of the same library type from the same tissue were available, these datasets were combined to increase read depth for *de novo* assembly (see Table S2). For paired end read datasets, only properly paired and singleton reads as defined by SAMTools were used.

#### Transcripts were filtered to remove overlap with non-lincRNA genes or pseudogenes and short transcripts

Transcripts less than 200 nt in length were removed. Remaining transcripts were removed if they were within 1 kb of RefSeq NM genes on the same strand or, in the case of transcripts with ambiguous strandedness, on either strand relative to the NM gene. Transcripts on the opposite strand of an NM gene were removed if they overlapped the NM gene by at least one base. In addition, transcripts overlapping at least one base of any of the following were removed, regardless of strandedness: Ensembl v61 genes except “lincRNA” and “processed_transcript”, non-human RefSeq genes aligned to hg18 with BLAT (UCSC Genome Browser “Other RefSeq” track), alternative and extended 5′ and 3′ UTRs of known human genes from UTRdb, RefSeq NR and XR transcripts annotated as “pseudogenes”, and Ensembl v54 coding sequences.

#### Transcripts containing large ORFs were removed

Two steps of filtering were performed to remove both putative protein coding transcripts and their UTRs. First, large ORFs (>100 amino acids) were identified in all transcripts in all reading frames using EMBOSS getorf v6.1.0. In order to account for potentially truncated ORF-containing transcripts in which the start or stop codon may be outside the annotated region, the presence of greater than 300 nt downstream of a start codon without an interrupting stop codon, or 300 nt upstream of a stop codon without an interrupting start codon, sufficed to call a putative ORF. Transcripts with putative large ORFs were removed. These putative large ORF containing intergenic transcripts, some of which may be novel protein coding genes, are provided as a resource in Dataset S10. In order to remove potential UTRs of these large ORF-containing transcripts from the lincRNA catalog, the remaining transcripts were filtered to remove any that overlapped a large ORF-containing transcript.

#### Transcripts overlapping extended protein coding gene structures were removed

RNA-seq reads may extend beyond annotated 5′ and 3′ ends of annotated protein coding gene structures representing possible extended UTRs as well as, in the case of spliced reads mapping to the gene from distal sites, unannotated exons. In order to avoid cataloging transcripts in these regions as lincRNAs, we created a filter based on extended boundaries of protein coding genes using RNA-seq data. To do this, *de novo* transcriptome assembly with Cufflinks v1.1.0 using RefSeq NM genes as a reference annotation (-g), upper quartile normalization (-N), and fragment bias correction (-b) was performed on all polyA+ RNA-seq libraries in Table S2. RefSeq NM gene annotations were used as the reference annotation for this transcript assembly because these represent a limited, high confidence set of protein coding gene annotations. This set of extended protein coding gene boundaries (Dataset S1) was used as a filter to remove transcripts that overlap any extended protein coding gene by at least one base regardless of strandedness.

#### Transcripts not expressed at FPKM>1 in at least one dataset were removed

In order to determine transcript expression levels, mapped RNA-seq reads were distributed to transcripts using a modified version of HTSeq v0.5.3p that allows for reads that are mapped to shared portions of overlapping transcripts to be counted as a full read for each overlapping transcript. This was necessary to properly assign reads to each of multiple redundant annotations of transcripts present in the combined set from all public databases and *de novo* assemblies prior to the merging of overlapping lincRNA annotations (described below). These redundant annotations are the result of the repeated *de novo* assembly of the same transcript in multiple different datasets or redundant existing annotations in public databases, each of which have slightly different genomic coordinates yet may represent the same transcript. As such, all reads were distributed fully to each redundant annotation rather than proportioned between them. Read counts were converted to FPKMs using total mapped reads for each dataset calculated by the SAMTools flagstat function and custom scripts. Transcripts not expressed at FPKM>1 in at least one dataset were removed. As a result of this FPKM>1 minimum filter, 99.975% of *de novo* assembled lincRNAs (pre-merging) have at least 5 reads supporting their expression in at least one of the combined datasets in Table S2, and >99.1% have at least 10 reads in one dataset. Transcripts were further categorized as FPKM>1, FPKM>10, and FPKM>30 in at least one dataset where each category is inclusive of all transcripts in higher categories.

#### Overlapping transcripts passing all filters at each expression cutoff were merged and grouped by proximity

To identify a minimal set of distinct lincRNAs, overlapping transcripts were merged if 50% of an exon of a transcript overlapped an exon of another transcript. Furthermore, merged transcripts within 1 kb of each other were placed in the same group but received distinct transcript numbers, and are named based on the FPKM expression level they were derived from, e.g. FPKM1_group_32871_transcript_1. Merging, grouping and naming was performed separately on all FPKM>1 transcripts, FPKM>10 transcripts, and FPKM>30 transcripts. Filtering statistics are presented in Table S3. The catalog of merged lincRNAs at each expression cutoff is in BED format for genome build hg18 in Dataset S2. The FPKM>1 catalog of lincRNAs was used for all analyses in this study unless stated otherwise. The lincRNA annotations are provided as BED files in the hg18 genome annotation rather than hg19 because the UCSC Genome Browser currently has more data “tracks” available for hg18. However, the lincRNA annotations may be readily converted to hg19 or other genome annotations by users with the LiftOver tool: http://genome.ucsc.edu/cgi-bin/hgLiftOver.

After merging these expression filtered, overlapping lincRNAs, FPKMs were recalculated (Dataset S8) for the merged lincRNAs using the modified HTSeq program described above. Due to the incomplete nature of the lincRNA structures resulting from *de novo* assembly, overlapping and nearby lincRNAs were considered to represent different potential models of the same lincRNA gene (rather than isoforms). Therefore, in the rare instances where two or more lincRNA models partially overlap but do not satisfy our merging criteria (above), the reads mapping to these overlapping portions were fully assigned to each lincRNA.

#### Identifying lincRNAs expressed significantly above other intergenic regions

For each RNA-seq dataset (Table S1), an empirical background distribution of expression values was generated using one million size-matched annotations shuffled randomly across intergenic sequence. The intergenic sequence used includes all portions of the uniquely mappable genome excluding RefSeq NM, NR and XR genes, Ensembl v61 genes including “lincRNAs” and “processed transcripts”, GENCODEv6 genes including “lincRNAs” and “processed transcripts”, H-Invitational “noncoding” transcripts, alternative and extended 5′ and 3′ UTRs of known human genes from UTRdb, extended protein coding gene structures derived from RNA-seq data (extended gene filter, described above), and published lincRNAs from Khalil *et al.* [18] and Cabili *et al.* [16]. To determine which putative lincRNAs (in Dataset S2, FPKM>1) were expressed significantly above background in at least one dataset the probability of observing a transcript at any given expression level was estimated using the dataset-specific background distribution and adjusted for multiple testing according to the Bonferroni correction assuming one test per RNA-seq dataset. Those lincRNA annotations with a corrected *P* value < = 0.1 in at least one dataset are cataloged in Datasets S6, S7.

### Additional LincRNAs Only Expressed in Cabili et al. [16]

An additional set of annotated lincRNA transcripts from Cabili *et al.* [16] passed all our filters except were not expressed at FPKM>1 in any of the datasets analyzed here and were therefore removed from the lincRNA catalog in Dataset S2. However, some of these transcripts were reported as expressed at FPKM>1 in at least one of the datasets analyzed in Cabili *et al.* [16], all of which are distinct from the datasets analyzed here. These additional lincRNAs were merged with the lincRNAs in the catalog in Dataset S2 resulting in an additional 920 lincRNAs in 741 groups at FPKM>1, 88 lincRNAs in 82 groups at FPKM>10, and 17 lincRNAs in 17 groups at FPKM>30. This expanded lincRNA catalog is in BED format for genome build hg18 in Dataset S3 and was not used further for any analyses in this study.

### Note on Genomic DNA Contamination in RNA-seq Datasets

Genomic DNA contamination is a potential source of false positive expression signal in RNA-seq data that may contribute to *de novo* assembly of erroneous transcripts. In principle, only exon-exon junction spanning reads can be unequivocally determined as derived from RNA. Proper *de novo* assembly of both nonspliced and spliced (aside from the exon-exon junction spanning reads) transcripts may therefore suffer if significant genomic DNA contamination is present. Because our analysis utilized a wide range of novel and previously existing RNA-seq datasets of unknown genomic DNA contamination content, we took multiple steps to mitigate this possibility. First, for all RNA-seq datasets, we analyzed the distribution of reads between protein coding exons compared to other regions with the expectation that read distributions should be similar between RNA-seq datasets generated from libraries of the same type (e.g. polyA+ selected). A dataset with an unusually high percentage of intronic and intergenic reads could contain significant genomic DNA contamination. Our analysis of the datasets used in this study revealed that, as expected, polyA+ specific RNA-seq datasets have a higher fraction of reads mapping to protein coding gene exons than rRNA-depleted or polyA− specific datasets. Furthermore, no obvious outlier datasets were found for any of the library types. The results of this analysis ensured that no datasets with high genomic DNA contamination were used in this study (Figure S2). Next, as described in Figure 2A and in the Methods, we applied both size and expression cutoffs for all lincRNAs. The size cutoff prevents miscalling errant single reads, either from genomic DNA contamination or from read mapping artifacts, as lincRNAs while the expression cutoff removes lincRNAs that are assembled from rare genomic DNA-derived reads. The combination of these approaches served to minimize the contribution of genomic DNA to the lincRNA catalog.

### Analysis of Distribution of LincRNAs Between Polyadenylated and Nonpolyadenylated RNA-seq Data

H9 ESC and HeLa RNA-seq data from fractions exclusively containing polyA− or polyA+ transcripts were analyzed [46]. Transcripts with RNA-seq reads in all four datasets and with FPKM>1 in at least one of the two fractions for each cell type were analyzed for Figure 3B (16,819 NM genes and 127 lincRNAs). For Figure S5, transcripts with reads in both fractions and FPKM>1 in at least one of the two fractions for a specific cell type were included in the analysis of that cell type (20,470 NM genes and 849 lincRNAs in H9 ESCs; 18,294 NM genes and 1,009 lincRNAs in HeLa). The whiskers of the box and whisker plot extend to +/−1.5 times the interquartile range or the most extreme datapoint.

### Paired-End Ditag (PET) Cluster Analysis

Publicly available paired-end ditag (PET) cluster annotations derived from 7 cell lines or tissues, generated by the ENCODE project, were downloaded from http://genome.ucsc.edu/cgi-bin/hgFileUi?db=hg19&g=wgEncodeGisRnaPet. The PET cluster annotation files used were (by cell or tissue type):

A549 (wgEncodeGisRnaPetA549CellPapClusters.bedCluster),

H1_hESC (wgEncodeGisRnaPetH1hescCellPapClustersRep1.bed),

HeLa-S3 (wgEncodeGisRnaPetHelas3CellPapClustersRep1.bed),

IMR90 (wgEncodeGisRnaPetImr90CellPapClusters.bedCluster),

MCF-7 (wgEncodeGisRnaPetMcf7CellPapClusters.bedCluster),

Prostate (wgEncodeGisRnaPetProstateCellPapClustersRep1.bed),

SK-N-SH (wgEncodeGisRnaPetSknshCellPapClusters.bedCluster).

Further description of these PET clusters, including how the annotations were generated, is available at the UCSC Genome Browser site here http://genome.ucsc.edu/cgi-bin/hgTrackUi?hgsid=321010719&c=chr21&g=wgEncodeGisRnaPet. BEDTools was employed to compute overlap between lincRNA and RefSeq NM gene 5′ and 3′ ends and PET cluster 5′ and 3′ end ‘blocks’. In the case of ambiguous stranded lincRNAs, both potential orientations were allowed for determining overlap with the 5′ and 3′ ends of PET clusters.

### Ribosome Profiling Analysis

Ribosome profiling data and matched mRNA-seq data from HeLa cells corresponding to the experiments (mock transfected 12 hr time point) presented in Guo *et al.* [22] were downloaded from the NCBI GEO (GSE22004). The expression level of the filtered set of lincRNAs and of RefSeq NM transcripts was evaluated as above. The 803 lincRNAs expressed at an FPKM>1 and a sample of 1292 RefSeq NM transcripts expressed at an FPKM>1 (divided into their constituent CDS and 3′ UTR regions) were broken up into 30 bp windows with a 1 bp offset. A modified version of HTSeq (described above) was used to count reads aligning to each window for both RNA-seq and ribosomal profiling data. The ratio of ribosome-associated reads over mRNA-seq reads was evaluated for each window and the maximum ratio for a given transcript was taken as a measure of ribosome engagement. The whiskers of the box and whisker plot in Figure 2B extend to +/−1.5 times the interquartile range with outliers depicted as dots. Wilcoxon rank sum test was used to calculate *P* values.

### Computational Analysis of Coding Potential

The program PhyloCSF (9/16/2010 release) [40] was used to computationally evaluate the coding potential of the filtered lincRNA transcripts. A BED file describing these lincRNA transcripts as well as a random sample of 8310 RefSeq NM transcripts was loaded onto the Galaxy webserver (https://main.g2.bx.psu.edu/) and the tool ‘Stitch Gene Blocks’ was used to retrieve multiple alignment files with sequence entries for the following genome builds based on the 44 way Multiz alignment to hg18: hg18 panTro2 rheMac2 tarSyr1 micMur1 otoGar1 tupBel1 mm9 rn4 dipOrd1 cavPor3 speTri1 oryCun1 ochPri2 vicPac1 turTru1 bosTau4 equCab2 felCat3 canFam2 myoLuc1 pteVam1 eriEur1 sorAra1 loxAfr2 proCap1 echTel1 dasNov2 choHof1. Genome build names were converted to common names and PhyloCSF was run using the options –orf = StopStop3 and –frames = 6.

### Chromatin Modification Analysis

ChIP-seq data from IMR90 cells [28] was retrieved from the NCBI SRA (Table 1) and aligned to hg18 using Bowtie v0.12.7 allowing only uniquely mapped reads (-k 1). A modified version of HTSeq v0.5.3p (described above) was used to count reads mapping to lincRNAs and RefSeq NM genes. The ratio of IP reads to matched input control reads was used as the measure of ChIP signal. RNA-seq data from IMR90 cells [29] was also analyzed to obtain FPKM values for lincRNAs and RefSeq NM genes using the same procedure used for lincRNA discovery. The whiskers of the box and whisker plot extend to +/−1.5 times the interquartile range or the most extreme data point.

**Table 1:** Datasets used for chromatin modification analysis.

Mark	Sample ID	SRA File ID(s)
H3K4me3	214	SRR029610, SRR029618
H3K9me3	805	SRR037619
H3K36me3	214	SRR037546, SRR037550, SRR037553, SRR037592
H3k27me3	803	SRR037555, SRR037560
Input	803	SRR037639
Input	805	SRR037640
Input	214	SRR037634, SRR037635, SRR037636

### Tissue Clustering by LincRNA Expression

RNA-seq datasets from B cells, H1 ESCs, and brain (see Table S1) were clustered by lincRNA expression levels. LincRNAs with FPKM>10 in one or more of the 7 RNA-seq datasets analyzed in Figure 3B were used to generate the heatmap and dendrogram. These 7 datasets were chosen for this analysis because they have replicates from each tissue and have deep read counts for all replicates (Table S1), important features for accurate measurement of differential expression. Using Gene Cluster 3.0, FPKM values were log_2_ transformed and the genes (rows) and samples (columns) were normalized by multiplying each log_2_ transformed FPKM value by a scale factor such that the sum of the squares of the values in each row and column are 1.0. Euclidean distance using centroid linkage was calculated for all samples and the heatmap and dendrogram was generated with Java TreeView. Red corresponds to fully induced expression and blue corresponds to fully repressed expression.

### Conservation Analysis

Base-wise conservation scores (PhyloP score calculated with PHAST), based on the multiple alignment of placental mammal genomes, were downloaded from the UCSC Genome Browser. The 50 bp window in each lincRNA transcript with the highest average PhyloP score was identified. The process was repeated for RefSeq NM genes and a set of size-matched (to lincRNAs) repetitive elements from RepeatMasker (UCSC Genome Browser). PhyloP scores for the maximally conserved 50 bp windows of each lincRNA are listed in Table S4.

### SNP Analysis

#### Enrichment of trait-associated SNPs

A table containing all trait-associated SNPs with *P*<10^−8^ was downloaded from the NCBI dbGaP Association Results Browser (3,781 total trait-associated SNPs). Genomic coordinates of trait-associated SNPs were retrieved from dbSNP 130. To compare enrichment of trait-associated SNPs in lincRNAs versus background loci (nonexpressed intergenic regions), regions of the uniquely mappable genome longer than 200 bp that exclude all evidence of transcription (RNA-seq reads, RefSeq NM, NR and XR genes and pseudogenes, Ensembl v61 genes, GENCODE v10 genes, spliced ESTs, spliced H-Invitational cDNAs, 5′ and 3′ UTRs from UTRdb, extended RefSeq NM genes derived using reference annotation based *de novo* transcriptome assembly (see above and Dataset S1) and all lincRNAs) were compiled and served as background loci for this analysis. The number of tested SNPs on Illumina (Illumina 1M) and Affymetrix (Affymetrix SNP Array 6.0) SNP arrays was determined for RefSeq NM gene exons, lincRNA exons and background loci. The number of tested SNPs per platform was scaled by the fractional contribution of Illumina (58.6%) versus Affymetrix (41.4%) platforms to the full set of GWASs in the NHGRI GWAS catalog [1]. The number of trait-associated SNPs per tested SNP was then determined using this scaled number of tested SNPs. Fisher's exact test was used to calculate *P* values and error bars in Figure 4 represent 95% binomial proportion confidence intervals.

#### Common SNPs

A table containing all common SNPs (minor allele frequency >0.05) from HapMap release #27 was downloaded from the BioMart HapMap site (http://hapmap.ncbi.nlm.nih.gov/biomart/martview) and the number of common SNPs within RefSeq NM gene exons, lincRNA exons and background loci divided by the number of genomic bases in each of these categories was determined. Fisher's exact test was used to calculate *P* values and error bars in Figure S7 represent 95% binomial proportion confidence intervals.

## Supporting Information

Dataset S1 Extended protein coding gene boundary filter (BED format; hg18).
(TXT)

Dataset S2 Primary catalog of lincRNAs identified and analyzed in this study (53,864 FPKM>1, 3,676 FPKM>10, and 925 FPKM>30 transcripts) (BED format; hg18).
(ZIP)

Dataset S3 Catalog of lincRNAs in Dataset S2 after merging with additional lincRNAs found to be expressed at FPKM>1 exclusively in Cabili *et al.* [16] (54,784 FPKM>1, 3,764 FPKM>10, and 942 FPKM>30 transcripts) (BED format; hg18).
(ZIP)

Dataset S4 Catalog of lincRNAs in Dataset S2 (FPKM>1) that are spliced (4,576 transcripts) (BED format, hg18).
(TXT)

Dataset S5 Catalog of lincRNAs in Dataset S2 that are expressed at FPKM>1 in at least two RNA-seq datasets (26,455 transcripts) (BED format, hg18).
(TXT)

Dataset S6 Catalog of lincRNAs in Dataset S2 (FPKM>1) that are statistically significantly (p<0.1) expressed above a random sample of other size-matched intergenic regions in at least one RNA-seq dataset (5,267 transcripts) (BED format, hg18).
(TXT)

Dataset S7 RNA-seq dataset names, P values and FPKMs corresponding to each significantly expressed lincRNA in Dataset S6.
(TXT)

Dataset S8 RNA-seq FPKM and read counts for all lincRNAs (from Dataset S2, FPKM>1) and NM genes in all individual datasets (TXT). Please note that these are large files: the compressed FPKM file is 32 MB (94 MB uncompressed) and the compressed counts file is 7 MB (29 MB uncompressed).
(ZIP)

Dataset S9 GENCODEv6 “lincRNAs” and “processed transcripts” that were removed at each step of filtering. (A) Unfiltered GENCODEv6 “lincRNAs” and “processed transcripts” (39,472 transcripts) (BED format; hg18) (TXT). (B) GENCODEv6 “lincRNAs” and “processed transcripts” that overlap RefSeq NM (protein coding) genes by at least 1 base pair on either strand (27,267 transcripts) (BED format; hg18) (TXT). (C) GENCODEv6 “lincRNAs” and “processed transcripts” that overlap (see Methods) one or more elements of an expanded set of protein coding genes (UCSC, RefSeq, Ensembl, GENCODE), pseudogenes, UTRs (UTRdb), or non-lincRNA noncoding RNAs (33,245 transcripts) (BED format; hg18) (TXT). (D) GENCODEv6 “lincRNAs” and “processed transcripts” that passed the protein/pseudogene/non-lincRNA ncRNAs/<200 nt filter, but contain an ORF>100 amino acids in length (964 transcripts) (BED format; hg18) (TXT). (E) GENCODEv6 “lincRNAs” and “processed transcripts” that do not themselves contain an ORF>100 amino acids, but overlap another annotated or *de novo* lincRNA that contains an ORF>100 amino acids (2,700 transcripts) (BED format; hg18) (TXT). (F) GENCODEv6 “lincRNAs” and “processed transcripts” that passed the prior filters but overlap an extended protein coding gene structure (149 transcripts) (BED format; hg18) (TXT). (G) GENCODEv6 “lincRNAs” and “processed transcripts” passing all prior filters except not found expressed at FPKM>1 in any dataset (1,469 transcripts) (BED format; hg18) (TXT). (H) GENCODEv6 “lincRNAs” and “processed transcripts” passing all filters and expressed at FPKM>1 in at least one dataset (945 transcripts) (BED format; hg18) (TXT).
(ZIP)

Dataset S10 Catalog of intergenic transcripts containing ORFs longer than 100 amino acids (105,265 transcripts) (BED format; hg18).
(TXT)

Figure S1 Fraction of the human genome with mapped RNA-seq reads at varying minimum read thresholds. The 4.5 billion mapped reads from all 127 RNA-seq datasets were combined and aligned to the uniquely mappable portion of the human genome (see Methods). The fraction of the uniquely mappable genome with at least the minimum read threshold is plotted. The data does not plateau at low minimum read thresholds, indicating that deeper sequencing would result in a further increase in the fraction of genome covered. For split reads (reads spanning an intron), the intervening (intronic) sequence was either inferred to have been transcribed (Including Inferred Bases) or was not (Excluding Inferred Bases). At the 1 read minimum read count threshold, 67.1% and 78.9% of the genome have read coverage when excluding or including inferred bases, respectively.
(TIF)

Figure S2 Fraction of RNA-seq reads mapping to protein coding (RefSeq NM) gene exons versus intronic and intergenic regions for 127 RNA-seq datasets grouped by RNA-seq library type. Read counting was performed using a modified version of HTSeq v0.5.3p (see Methods). Isoforms of protein coding genes were flattened before reads were counted such that reads were distributed only once per gene even if multiple isoforms exist. PolyA+ selected libraries (enriched for mRNAs) contain a higher fraction of reads mapping to protein coding gene exons while ribosomal RNA-depleted RNA-seq libraries and polyA− selected libraries contain a higher fraction of intronic and intergenic reads. In all cases, due to the generally high expression levels of protein coding genes, protein coding gene exons contain a disproportionate number of mapped reads relative to the genomic space they occupy (<3%).
(TIF)

Figure S3 Fraction of lincRNAs (Dataset S2, FPKM>1) expressed at varying minimum FPKM levels. The fraction of lincRNAs in Dataset S2 that are expressed at or above the corresponding FPKM level in at least one dataset is plotted.
(TIF)

Figure S4 LincRNAs have tissue specific expression patterns. LincRNA expression levels (FPKMs) were used to cluster replicates of RNA-seq data from B cells, H1 embryonic stem cells and brain tissue. Agglomerative hierarchical clustering of both lincRNAs (rows) and samples (columns) by Euclidean distance was performed with log_2_ transformed lincRNA FPKM values for lincRNAs with FPKM>10 in at least one of the analyzed samples. The heatmap displays red for fully induced lincRNAs and blue for fully repressed lincRNAs, where rows and columns were normalized (see Methods).
(TIF)

Figure S5 Polyadenylation of lincRNAs versus protein coding genes. Distribution of ratios of FPKMs in polyA+/polyA− fractions for lincRNAs and NM genes in HeLa and H9 ESCs. Transcripts with reads in both fractions and FPKM>1 in at least one of the two fractions for a specific cell type were included in the analysis of that cell type (20,470 NM genes and 849 lincRNAs in H9 ESCs; 18,294 NM genes and 1,009 lincRNAs in HeLa). Whiskers extend to +/−1.5 times interquartile range or most extreme data point.
(TIF)

Figure S6 Comparison of conservation of the full lincRNA catalog (53,864 lincRNAs, Dataset S2, FPKM>1) to GENCODEv6 lincRNAs. The maximally conserved 50 bp windows in each lincRNA, RefSeq NM gene and repetitive element (nonconserved control sequences) were determined. Only the GENCODE lincRNAs that passed all lincRNA filters (2,414 GENCODE lincRNAs, Table S3) were evaluated.
(TIF)

Figure S7 Distribution of common SNPs between lincRNA exons, NM gene exons, and nonexpressed intergenic regions. HapMap II SNPs with minor allele frequency >0.05 located within NM gene exons, lincRNA exons, or background loci (nonexpressed intergenic regions), normalized by total number of base pairs in each region, were counted (*P = 0.0173, ** *P*<2.2E-16; error bars represent 95% binomial proportion confidence interval).
(TIF)

Table S1 Features of the RNA-seq datasets analyzed. P values correspond to a binomial test of proportion of the FPKM = 1 expression threshold in each dataset (see Methods).
(XLSX)

Table S2 Features of the combined RNA-seq datasets that were used for *de novo* transcriptome assembly.
(XLSX)

Table S3 LincRNA filtering statistics.
(XLSX)

Table S4 Conservation (PhyloP) score for the maximally conserved 50 bp window of each lincRNA in Dataset S2 (FPKM>1). 532 lincRNAs do not contain 50 contiguous bases with PhyloP scores and therefore are not listed.
(XLSX)

Table S5 Fraction of *de novo* assembled lincRNAs (pre-merging) discovered by *de novo* assembly in each combined dataset (see Table S2) that are spliced.
(XLSX)

Table S6 LincRNA (Dataset S2) and RefSeq NM gene analysis for experimental support of 5′ and 3′ end annotations using combined paired-end ditag (PET) data from 7 tissues/cell lines generated by the ENCODE project (see Methods).
(XLSX)
