# Supplementary figures and images for: Pervasive Transcription of the Human Genome Produces Thousands of Previously Unidentified Long Intergenic Noncoding RNAs
Source: PLoS Genet. 2013 Jun 20;9(6):e1003569. doi: 10.1371/journal.pgen.1003569 (PMC3688513; doi:10.1371/journal.pgen.1003569)

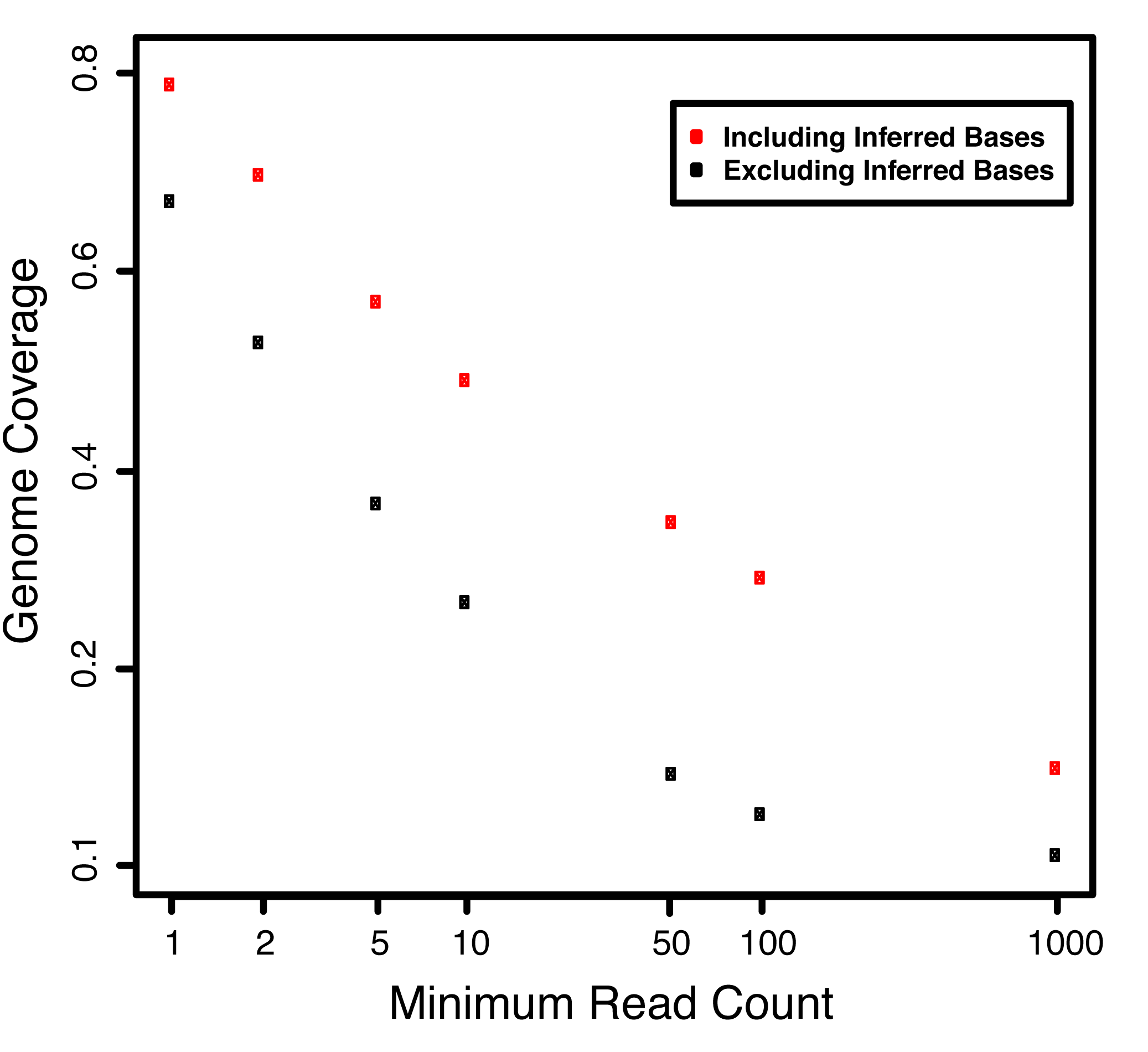

Supplement: Figure S1 — Fraction of the human genome with mapped RNA-seq reads at varying minimum read thresholds. The 4.5 billion mapped reads from all 127 RNA-seq datasets were combined and aligned to the uniquely mappable portion of the human genome (see Methods). The fraction of the uniquely mappable genome with at least the minimum read threshold is plotted. The data does not plateau at low minimum read thresholds, indicating that deeper sequencing would result in a further increase in the fraction of genome covered. For split reads (reads spanning an intron), the intervening (intronic) sequence was either inferred to have been transcribed (Including Inferred Bases) or was not (Excluding Inferred Bases). At the 1 read minimum read count threshold, 67.1% and 78.9% of the genome have read coverage when excluding or including inferred bases, respectively. (TIF) [file pgen.1003569.s011.tif]

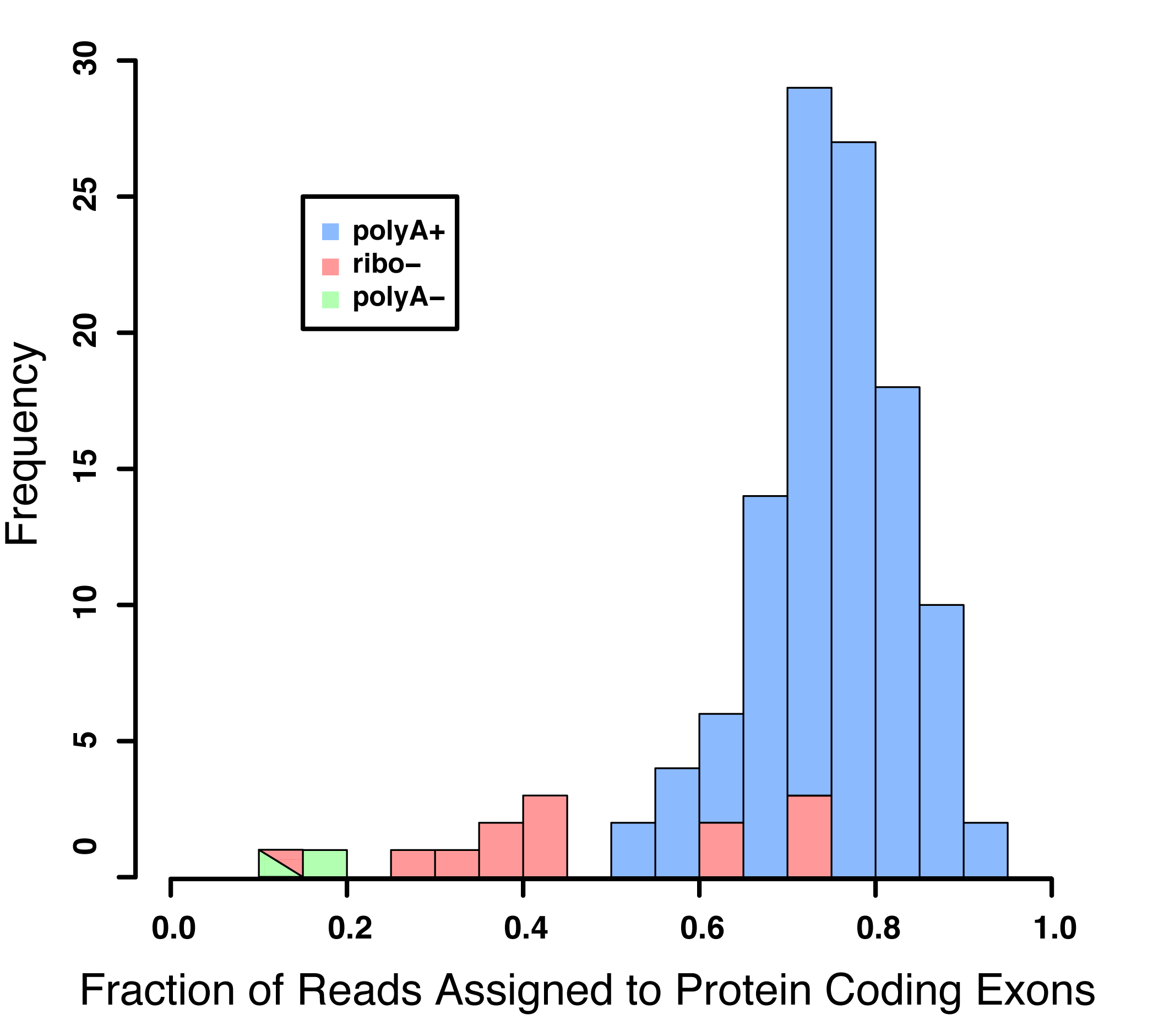

Supplement: Figure S2 — Fraction of RNA-seq reads mapping to protein coding (RefSeq NM) gene exons versus intronic and intergenic regions for 127 RNA-seq datasets grouped by RNA-seq library type. Read counting was performed using a modified version of HTSeq v0.5.3p (see Methods). Isoforms of protein coding genes were flattened before reads were counted such that reads were distributed only once per gene even if multiple isoforms exist. PolyA+ selected libraries (enriched for mRNAs) contain a higher fraction of reads mapping to protein coding gene exons while ribosomal RNA-depleted RNA-seq libraries and polyA− selected libraries contain a higher fraction of intronic and intergenic reads. In all cases, due to the generally high expression levels of protein coding genes, protein coding gene exons contain a disproportionate number of mapped reads relative to the genomic space they occupy (<3%). (TIF) [file pgen.1003569.s012.tif]

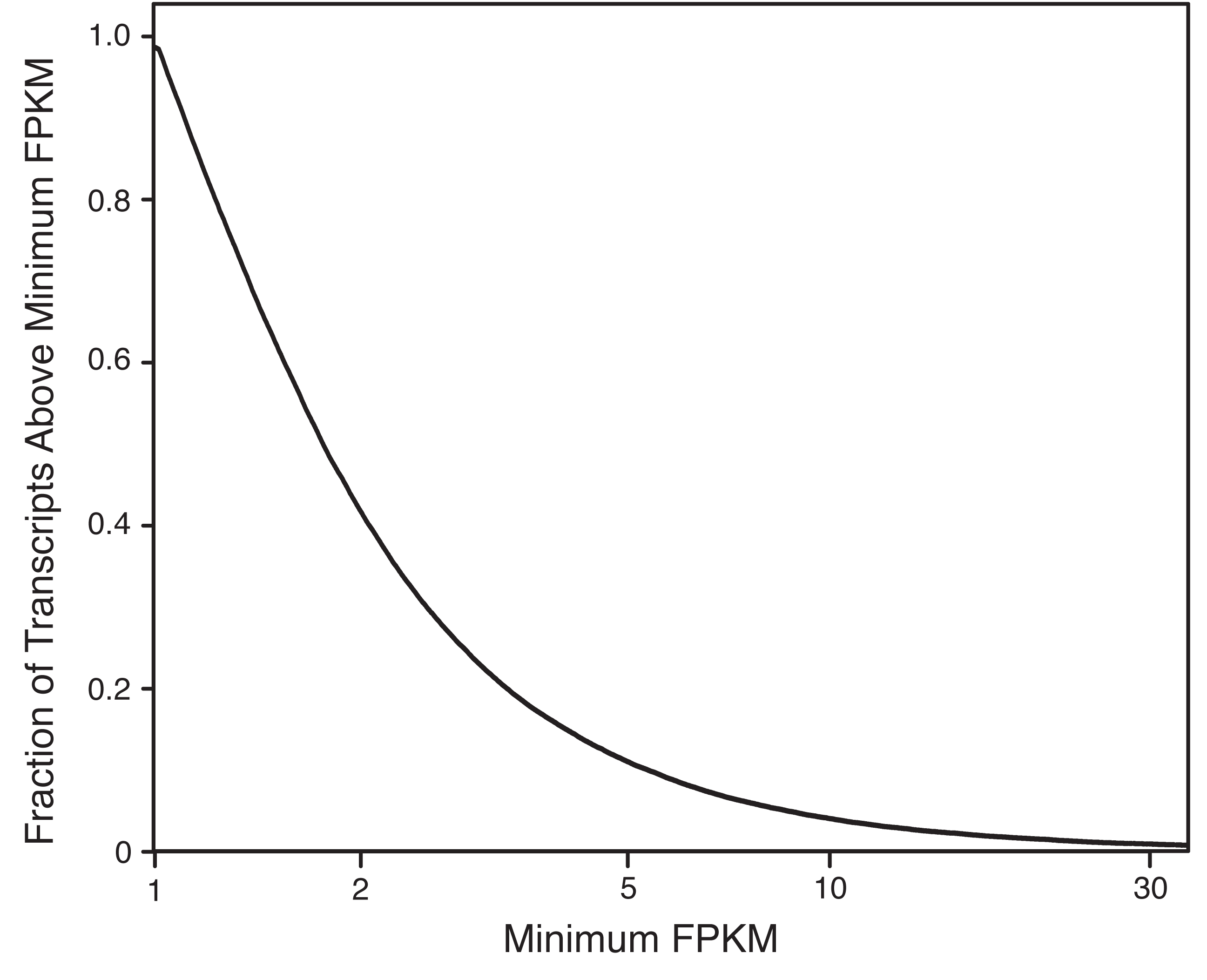

Supplement: Figure S3 — Fraction of lincRNAs (Dataset S2, FPKM>1) expressed at varying minimum FPKM levels. The fraction of lincRNAs in Dataset S2 that are expressed at or above the corresponding FPKM level in at least one dataset is plotted. (TIF) [file pgen.1003569.s013.tif]

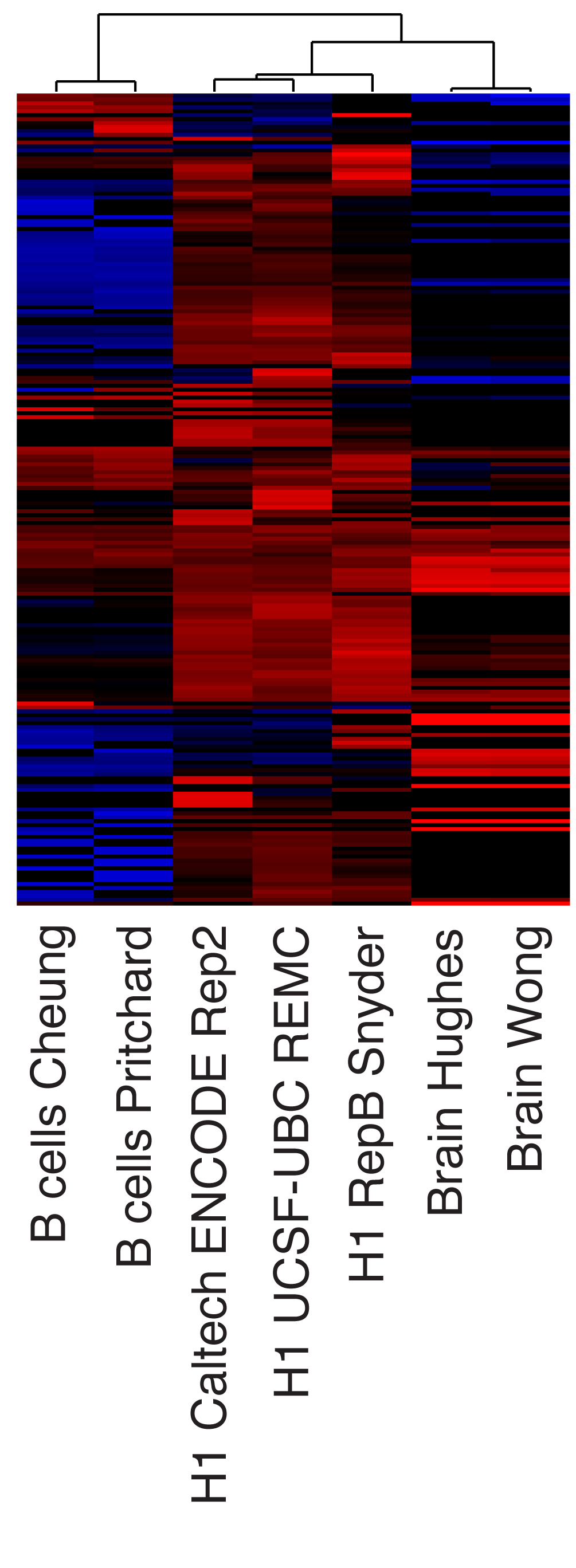

Supplement: Figure S4 — LincRNAs have tissue specific expression patterns. LincRNA expression levels (FPKMs) were used to cluster replicates of RNA-seq data from B cells, H1 embryonic stem cells and brain tissue. Agglomerative hierarchical clustering of both lincRNAs (rows) and samples (columns) by Euclidean distance was performed with log2 transformed lincRNA FPKM values for lincRNAs with FPKM>10 in at least one of the analyzed samples. The heatmap displays red for fully induced lincRNAs and blue for fully repressed lincRNAs, where rows and columns were normalized (see Methods). (TIF) [file pgen.1003569.s014.tif]

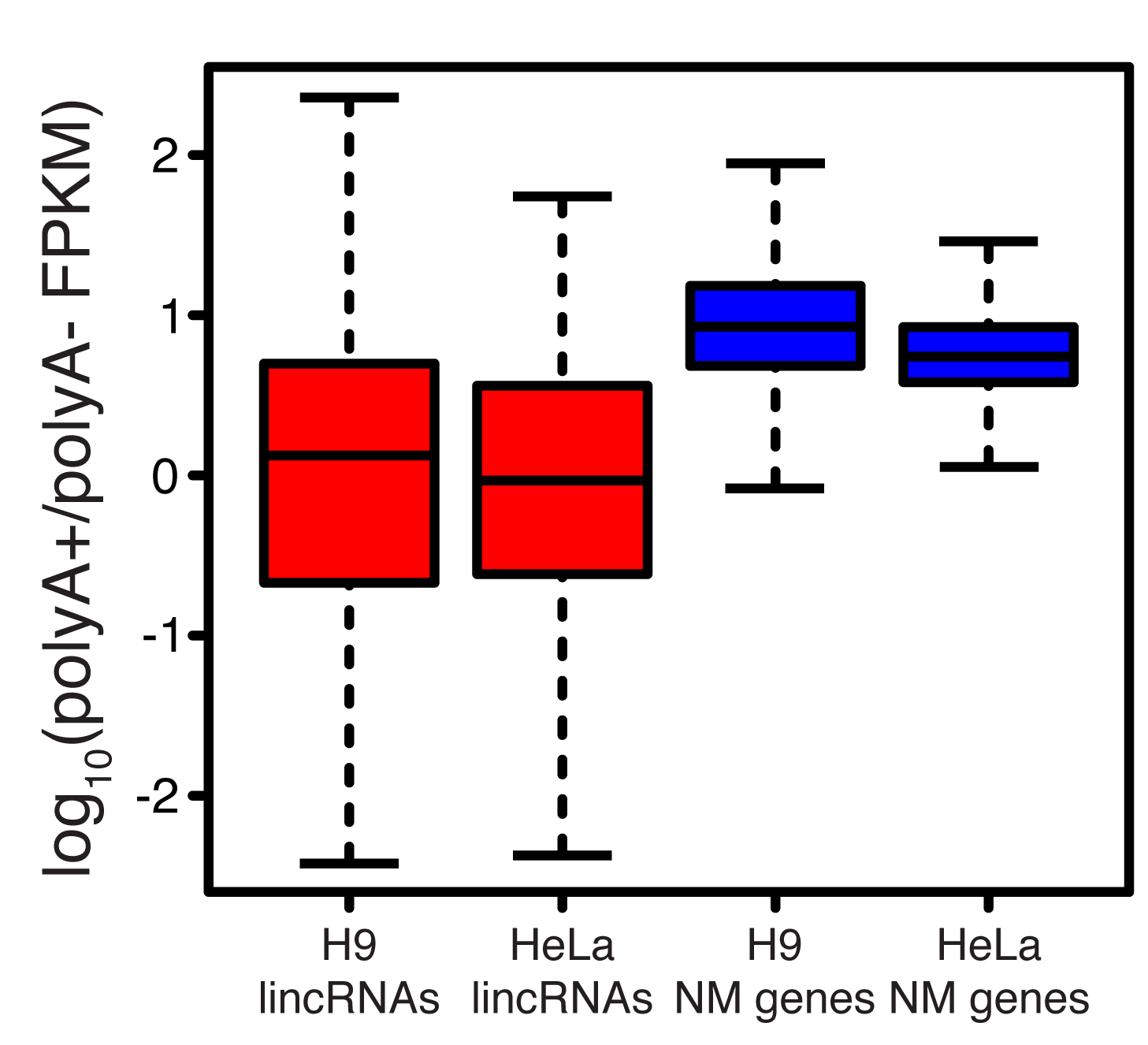

Supplement: Figure S5 — Polyadenylation of lincRNAs versus protein coding genes. Distribution of ratios of FPKMs in polyA+/polyA− fractions for lincRNAs and NM genes in HeLa and H9 ESCs. Transcripts with reads in both fractions and FPKM>1 in at least one of the two fractions for a specific cell type were included in the analysis of that cell type (20,470 NM genes and 849 lincRNAs in H9 ESCs; 18,294 NM genes and 1,009 lincRNAs in HeLa). Whiskers extend to +/−1.5 times interquartile range or most extreme data point. (TIF) [file pgen.1003569.s015.tif]

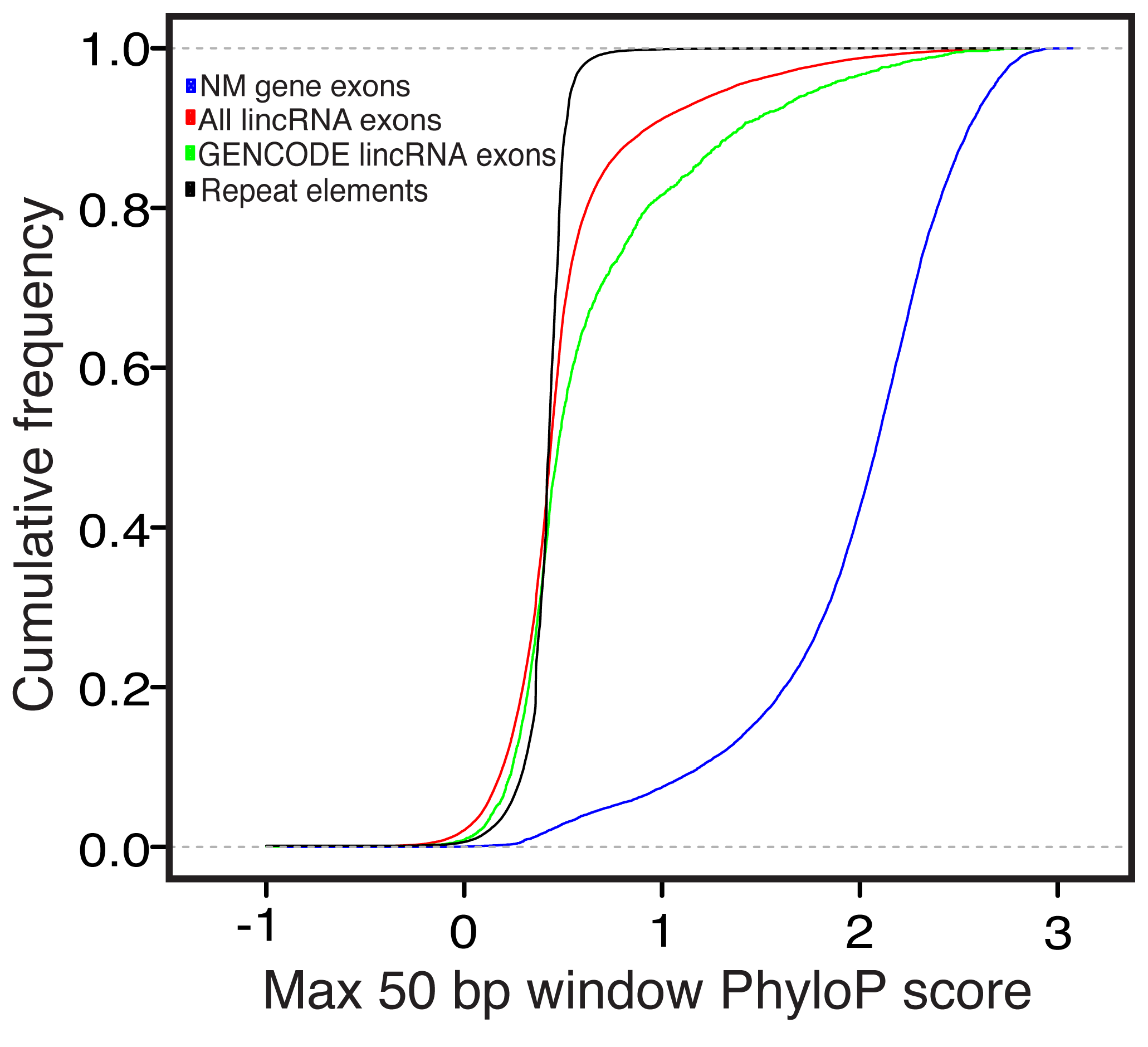

Supplement: Figure S6 — Comparison of conservation of the full lincRNA catalog (53,864 lincRNAs, Dataset S2, FPKM>1) to GENCODEv6 lincRNAs. The maximally conserved 50 bp windows in each lincRNA, RefSeq NM gene and repetitive element (nonconserved control sequences) were determined. Only the GENCODE lincRNAs that passed all lincRNA filters (2,414 GENCODE lincRNAs, Table S3) were evaluated. (TIF) [file pgen.1003569.s016.tif]

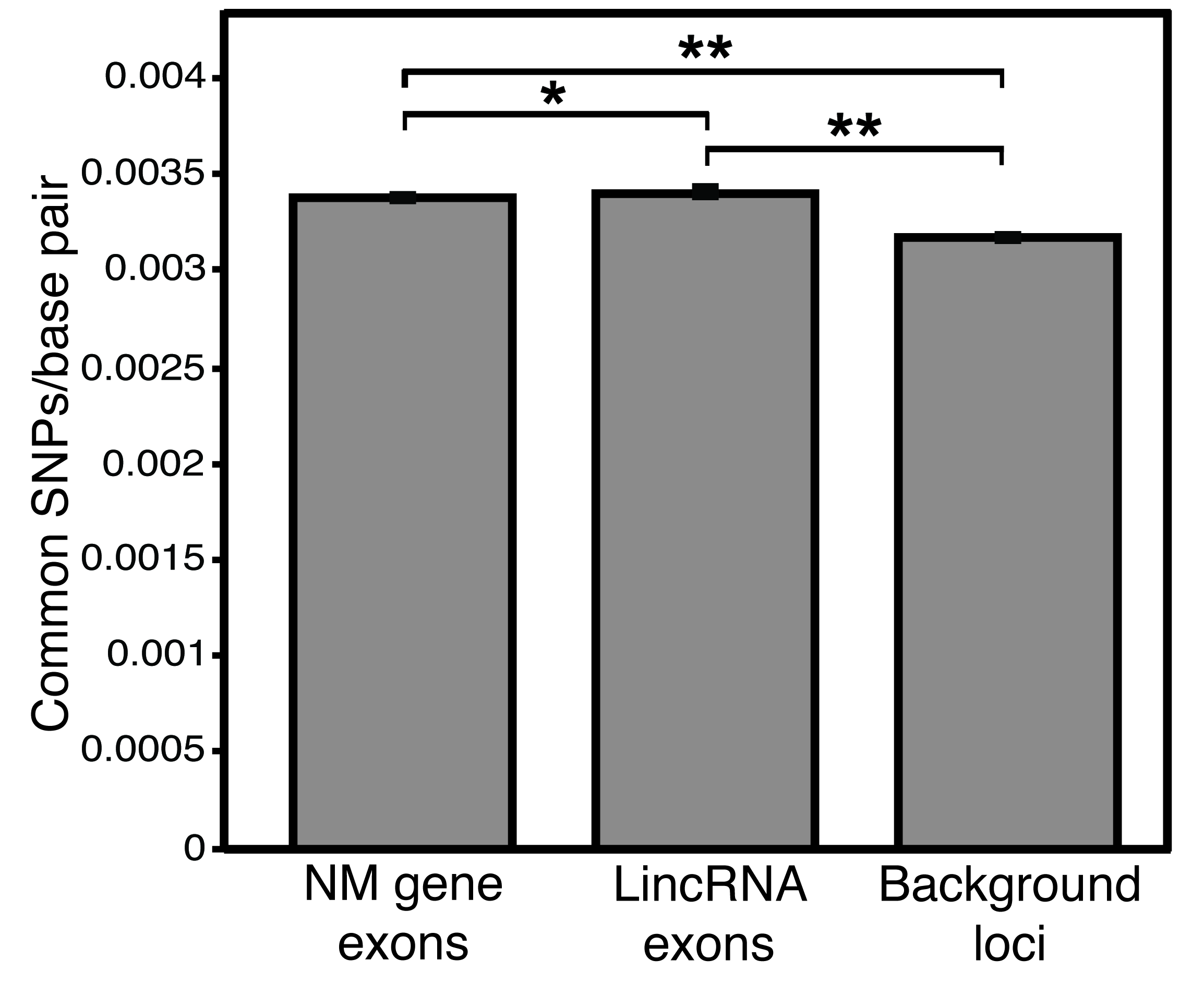

Supplement: Figure S7 — Distribution of common SNPs between lincRNA exons, NM gene exons, and nonexpressed intergenic regions. HapMap II SNPs with minor allele frequency >0.05 located within NM gene exons, lincRNA exons, or background loci (nonexpressed intergenic regions), normalized by total number of base pairs in each region, were counted (*P = 0.0173, ** P<2.2E-16; error bars represent 95% binomial proportion confidence interval). (TIF) [file pgen.1003569.s017.tif]
